# Complete resection of undifferentiated cardiac sarcoma and reconstruction of the atria and the superior vena cava: case report

**DOI:** 10.1186/1749-8090-7-96

**Published:** 2012-09-27

**Authors:** Nobuyuki Furukawa, Jan Gummert, Jochen Börgermann

**Affiliations:** 1 Department of Cardiothoracic Surgery, Heart and Diabetes Center North Rhine-Westphalia, Georgstr. 11, 32545, Bad Oeynhausen, Germany

**Keywords:** Cardiac sarcoma, Primary cardiac tumor

## Abstract

Primary cardiac tumors are rare with an incidence ranging from 0.001% to 0.03% in autopsy series. The prognosis of cardiac sarcomas remains poor because it proliferates rapidly and distant metastases are often found at diagnosis. A 47-year-old male complained of persistent cough. The chest roentgenogram was normal. Subsequent computed tomography revealed a mass in the right atrium. Echocardiography and magnetic resonance imaging confirmed also a right atrial mass (34 x 35 mm) infiltrating the atrial septum. The tumor was completely resected en bloc, including the anterior and lateral right atrial walls, the left atrial dome, and a large segment of the superior vena cava, and reconstructed the atria and superior vena cava with bovine pericardium. The tumor was histologically and immunohistochemically diagnosed as undifferentiated pleomorphic sarcoma. This type of cardiac sarcoma is very rare and usually found in the left atrium. Twenty-seven months after surgery, the patient is doing well without metastasis or local tumor recurrence.

## Background

Primary cardiac tumors are uncommon (incidence in autopsy series, 0.001–0.03%). Among them, malignant neoplasms account for <25% [1]. The prognosis of cardiac sarcomas remains poor. Although a more effective therapy would be desirable, complete resection is the current standard treatment in cases without metastases. We describe a case of undifferentiated pleomorphic sarcoma infiltrating both atria and the superior vena cava. We removed the tumor completely, including the sinus node, by en bloc resection and reconstructed the atria and superior vena cava with bovine pericardium.

## Case Presentation

**Figure 1:**
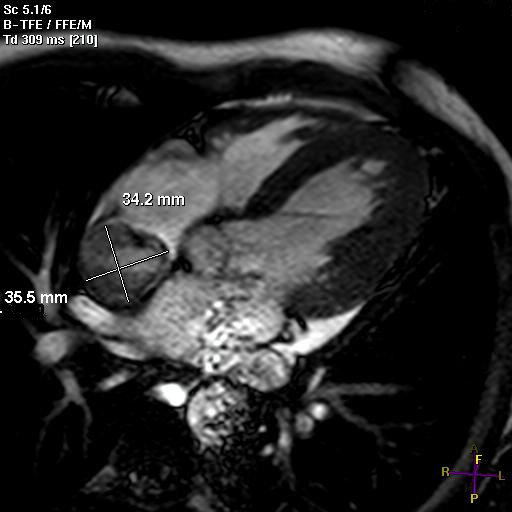
**Cardiac magnetic resonance imaging demonstrating a mass in the right atrium involving the left atrial dome and atrial septum**.

A 47-year-old male complained of persistent cough. Laboratory tests showed a high CRP level and an elevated erythrocyte sedimentation rate. The patient had a medical history of thalassemia minor and mild anemia but was not taking any medication. Physical examination and chest roentgenogram were unremarkable. Electrocardiography revealed sinus rhythm and incomplete right bundle branch block. Subsequent computed tomography demonstrated a right atrial mass, but no other anomalies. Echocardiography and magnetic resonance imaging confirmed a right atrial mass (34 × 35 mm) suspicious for a malignant cardiac tumor infiltrating the atrial septum (Figure 1). The tricuspid valve was intact; right atrial and ventricular sizes were normal; and left ventricular function was good.

**Figure 2:**
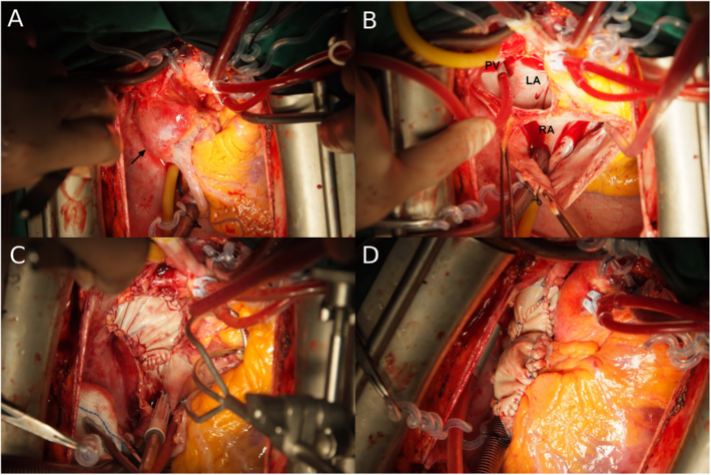
**Intraoperative view.** Note that the tumor extends into an through the wall of the superior vena cava and the left atrial dome (**A**). Complete tumor removal by en bloc resection, including the right atrial anterior and lateral wall, left atrial dome, and a large segment of the superior vena cava (**B**). Repair of the left atrial dome (**C**). Reconstruction of the right atrium and superior vena cava with bovine pericardium (**D**).

The patient was referred to our institution for treatment. The heart was exposed via mid-sternotomy. Because of severe narrowing from the tumor, the superior vena cava was unsuitable as a cannulation site. After full heparinization and flooding of the surgical field with CO_2_, the ascending aorta, inferior vena cava, and the innominate vein were cannulated. Cardiopulmonary bypass was commenced after the target ACT ≥ 450s was reached. A vent was placed into the left ventricle. Exploration revealed that the tumor was penetrating the walls of the right atrium and of the superior vena cava (Figure 2A). On complete bypass, the ascending aorta was crossclamped and multiple doses of warm blood Calafiore cardioplegic solution were instilled via the aortic root. The tumor was resected en bloc, including the anterior and lateral right atrial walls (approximately two thirds of the right atrium altogether), the left atrial dome, and a large segment of the proximal superior vena cava (Figure 2B). The atrial septum was found to be free. The left atrial roof was reconstructed with a patch of bovine pericardium (Figure 2C) secured with running 4–0 prolene (Ethicon, Norderstedt, Germany). The right atrium and the superior vena cava were each reconstructed with a folded strip of bovine pericardium, again using running 4–0 prolene suture, making sure that the neo-right atrial dome provided sufficient reservoir capacity (Figure 2D). The preliminary histologic evaluation revealed clear margins. The aortic clamp was removed after careful de-airing. As expected, the heart was beating in a nodal escape rhythm because the sinus node had been removed. We therefore secured permanent bipolar epicardial pacemaker leads to the remaining right atrium and to the left ventricle. The leads were brought out through the inner layers of the thoracic wall and connected to a DDD pacemaker which was implanted in the left pectoral area. Under mild inotropic support, the patient was easily weaned from cardiopulmonary bypass. All cannulas were removed and the cannulation sites oversewn. Protamin was administered. The pericardium was loosely approximated over a drain. The wound was closed in layers after careful hemostasis. The patient was transferred to the intensive care unit in stable condition.

The patient recovered without complications, was discharged home on postoperative day 7 and referred to undergo local radiotherapy and systemic chemotherapy with ifosfamide and paclitaxel. For a duration of three months, the patient was anticoagulated with warfarin and then switched to oral acetyl salicylic acid. Thirty-five months after surgery, the patient is doing well and remains free of metastases or local tumor recurrence.

**Figure 3:**
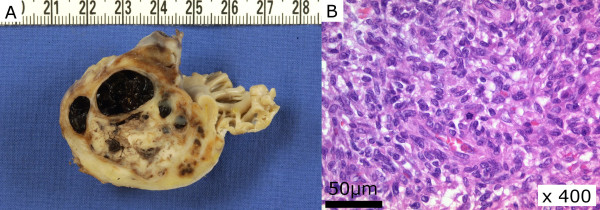
**Cut surface of the lesion showing the pale yellow and solid part of the tumor and the cystic component which is filled with coagulated blood.** (**A**) An HE-stained microscopic section reveals dense groups of polygonal and spindle cells with eosinophilic cytoplasm and large pleomorphic nuclei with high density chromatin and irregular nucleoli (**B**).

Histological examination (Figure 3) revealed dense groups of polygonal and spindle cells with eosinophilic cytoplasm and large pleomorphic nuclei with high density chromatin and irregular nucleoli. On immunohistochemical examination, the neoplastic cells stained positive for vimentin but negative for pan-keratin, desmin, actin, and histiocyte markers. These observations are compatible with a histological diagnosis of undifferentiated pleomorphic sarcoma.

## Discussion

Primary cardiac tumors are uncommon. Approximately three fourth of them are malignant [1]. Sarcomas comprise two-thirds of these malignancies [2]. Cardiac sarcomas include angiosarcoma; sarcoma with myofibroblastic differentiation; synovial sarcoma; and rhabdomyosarcoma. Sarcomas with myofibroblastic differentiation are the most diverse types, subclassified as undifferentiated pleomorphic sarcoma; osteosarcoma; leiomyosarcoma; and fibrosarcoma [3]. In several large series [4,5], the most common type was angiosarcoma, while undifferentiated pleomorphic sarcomas were relatively rare (only 6–7%). Undifferentiated pleomorphic sarcoma is usually found in the left atrium and exhibits a solid and infiltrative pattern. As described here, undifferentiated pleomorphic sarcoma with bilateral involvement is very rare [6].

The prognosis of patients with cardiac sarcoma is poor because the tumor proliferates rapidly and distant metastases are often found at the time of the initial diagnosis. Hamidi *et al*. reported that the median survival of patients who underwent surgery was 12 months, whereas survival of patients not operated upon was only 1 month [1]. Simpson *et al*. reported that the median survival of patients with and without metastasis was 5 and 15 months, respectively [4].

In our case, the tumor infiltrated the left atrium and approximately two-thirds of the right atrium, involving the sinus node and superior vena cava. Fortunately, this infiltration was relatively anterior in the left atrial dome, facilitating complete resection and repair with bovine pericardium. When the tumor infiltrates the left atrium more deeply and to a greater extent, resection and reconstruction is relatively difficult. In such cases, autotransplantation is an option permitting complete resection and repair [2,7].

Cardiac sarcomas are often asymptomatic. In advanced cases, they occasionally produce nonspecific symptoms. Malignant cardiac tumors with direct invasion of the pericardium can cause pericardial effusion or arrhythmias, such as atrial fibrillation, heart block, or recurrent ventricular tachycardia [2,3]. We performed en-bloc resection of the tumor, including the sinus node. Simultaneously, permanent epicardial pacemaker leads were placed onto the remaining right atrium and onto the left ventricle. Radical resection of the tumor is difficult or impossible if the tumor infiltrates the conduction system [8]. In many cases, cardiac sarcoma is so widespread that only palliative surgery can be performed [8]. It remains unclear if there is an alternative to radical surgical resection in patients without metastatic disease. Because cardiac sarcoma is uncommon and because there are only a few limited studies on the subject, no standard treatment has been defined. Although aggressive resection of cardiac sarcoma is challenging, some studies clearly demonstrate that complete tumor removal is the best method for improving survival [1,2,4].

In our case, chemotherapy and radiotherapy were initiated relatively early after surgery, especially in view of reports [2,7] of local recurrences or distant metastases several years after successful resection. The effectiveness of such adjuvant therapies for cardiac sarcoma remains controversial. However, studies [1,2] have shown that combining several carcinostatics is more effective than single-agent therapy, and that postoperative radiotherapy can improve survival.

## Conclusion

We presented a rare case of undifferentiated pleomorphic cardiac sarcoma successfully removed by en-bloc resection which included two-thirds of the right atrium, the left atrial dome and a large segment of the superior vena cava, reconstructing the defects with bovine pericardium. Despite successful management, close follow-up is mandatory because of the overall poor prognosis of cardiac sarcoma.

## Consent

Written informed consent was obtained from the patient for publication of this case report and any accompanying images. A copy of the written consent is available for review by the Editor-in-Chief of this journal.

## Competing interests

The authors declare that they have no competing interests.

## Authors’ contributions

NF carried out the manuscript and collected references. JB and JG helped to revise the manuscript. JB and JG underwent the operation. All authors read and approved the final manuscript.
